# IAIS Symposium on Novel Molecular Approaches to Anti-inflammatory Therapy 22–23 July 1994 Toronto Canada

**DOI:** 10.1155/S0962935194000426

**Published:** 1994


Mediators of Inflammation 3, 303–311 (1994)

IUPHAR 1994

### IAIS Symposium on Novel Molecular Approaches to Anti-inflammatory Therapy 22–23 July 1994 Toronto Canada

### Abstracts

### Role of protein tyrosine phosphorylation on COX-2 induction in endotoxin-activated endothelial cells and macrophages: a comparison with nitric oxide synthase

*P. Akarasereenont, J. A. Mitchell, I. Appleton, C. Thiemermann and J. R. Vane*
The William Harvey Research Institute,
St Bartholomews' Hospital Medical College,
Charterhouse Square, London EC1M 6BQ, UK

Cyclooxygenase (COX) and nitric oxide synthase (NOS) are two enzymes which have distinct cytokine-inducible isoforms (COX-2 and iNOS, respectively). Many cytokine receptors have an intracellular tyrosine kinase domain. Here, we have used the tyrosine kinase inhibitors, erbstatin and genistein, to investigate the potential role of tyrosine kinase activation in the induction of COX-2 and iNOS caused by endotoxin (lipopolysaccharide; LPS at 1 μg/ml for 24 h) in bovine aortic endothelial cells (BAEC) and J774.2 macrophages. The predominant COX metabolites, 6-oxo-prostaglandin (PG) $F_{1\alpha}$ (for BAEC) and $PGF_{2\alpha}$ (for 774.2 macrophages) were measured by radioimmunoassay under the following experimental conditions: (i) accumulation of COX metabolites from endogenous arachidonic acid was measured at 24 h after addition of LPS (1 μg/ml); (ii) in experiments designed to measure 'COX activity', COX metabolites generated by LPS-activated BAEC or J774.2 macrophages were assayed after incubation with exogenous arachidonic acid (30 μM for 15 min). Western blot analysis with a specific antibody to COX-2 was used to determine the expression of COX-2 protein caused by LPS in cell extracts. Accumulation of nitrite (measured by the Griess reaction) was used to measure NOS activity. Erbstatin (0.05–5 μg/ml) or genistein (0.5–50 μg/ml) caused a dose-dependent inhibition of the accumulation of COX metabolites in the supernatant of LPS-activated BAEC or J774.2 macrophages. Erbstatin or genistein also caused a dose-dependent inhibition of 'COX activity' in both cell types. Western blot analysis showed that erbstatin (5 μg/ml) or genistein (50 μg/ml) inhibited the expression of COX-2 protein in LPS activated BAEC and J774.2 macrophages. Erbstatin or genistein also caused a dose-dependent inhibition of nitrite accumulation in J774.2 macrophages activated with LPS (1 μg/ml for 24 h). In contrast to J774.2 macrophages, BAEC stimulated with LPS did not produce detectable amounts (< 1 μM) of nitrite. These results show that tyrosine phosphorylation is part of the signal transduction mechanism that mediates (i) the induction of COX-2 and iNOS elicited by LPS in J774.2 macrophages, and (ii) the induction of COX-2 by LPS in BAEC.

### Inhibition of the local Shwartzman reaction in rabbits by CSVTCG, a thrombospondin-derived hexapeptide

*C. E. Burrowes, S. Peers, L. Tran, and B. Beaubien*
Allelix Biopharmaceuticals Inc., 6850 Goreway Drive,
Mississauga, Ontario, Canada L4V 1P1

Thrombospondin (TSP) is a multidomain protein involved in cell adhesion processes through its associations with CD36 (GPIV, GPIIIb), $\alpha_v\beta_3$, $\alpha_4\beta_1$, and perhaps other binding proteins. The CSVTCG motif in TSP is responsible for TSP binding to CD36, and is reported to mediate tumour cell adhesion and platelet aggregation. The peptide form of CSVTCG blocks TSP-induced cell–cell associations. We were interested in determining whether this peptide might reduce microvascular pathophysiology, and thus tested it in a model of dermal thrombo-haemorrhage, the local Shwartzman reaction (LSR), in rabbits. The LSR was produced by intradermal injection of endotoxin (preparative dose) followed by intravenous administration of endotoxin (provocative dose) 18–24 h later. Haemorrhagic lesions that developed in the prepared skin sites were quantified by $^{111}$In-labelled erythrocytes. CSVTCG, at a dose of 1.0 mg/kg, given i.v. 15 min before the provocative dose induced a dose-dependent inhibition of haemorrhage (max. 80%, $p < 0.05$). The same dose of CSVTCG given i.v. 15 min before the preparatory dose was less effective and, when given i.d., had no effect on haemorrhage. These *in*

© 1994 Rapid Communications of Oxford Ltd

*vivo* experiments indicate that TSP is involved in thrombo-haemorrhagic events.

### Telemetric study of monoiodocetate (MIA) induced osteoarthritis in rats

*D. Chevrier, P. Gegout, C. Guingamp, P. Gillet, B. Terlain and P. Netter*
Department of Pharmacology, URA CNRS 1288, BP184, 54505 Nancy, France

Patients' handicap is the main point during osteoarthritis (OA). Telemetry, in automatically recording mobility of laboratory animals, allows measurement of incapacity during experimentally induced OA, especially in rats. As surgical models are inadapted to telemetry, and after preliminary studies of the impact of various chemical agents on rats' mobility, spontaneous nocturnal activity was recorded in Wistar rats after i.a. injection of MIA in both knees.

As a result, nocturnal mobility was significantly reduced in the MIA group (3 mg) when compared with controls (saline i.a.). Typically, MIA-induced incapacity advanced in three phases: day 1–3 marked and acute fall; day 4–14: recovery; then from day 15, progressive incapacity. This secondary handicap appears very similar to that observed in human disease; late and slowly progressive oncoming, moderate amplitude, aggravation without related inflammatory phenomenons (lack of fever and histological signs). Morphological aspect is in favour of an OA process, showing cartilate erosions and intense osteophytic proliferation. $^{35}S$ incorporation was assessed *ex vivo* in a centropatellar punch and in the remaining patella. Loss of centropatellar $^{35}S$ incorporation seems correlated with the loss of mobility (similar evolution in three phases). Contrasting with this decrease of anabolism in the central punch, the surrounding cartilage exerted an increased anabolism (osteophytes formation). Early, but not late administration, of indomethacin (3 mg/kg/day) reinforced hypoanabolism in the punch.

Thus, MIA-induced incapacity mimics human OA. As a predictible factor, handicap profile allows studies of early markers of OA detectable before installation of a marked incapacity. In addition, effects of treatments can be studied depending whether administrated before or after the onset of this handicap. Further explorations on relationships between handicap and biochemical changes might lead to new pharmacological targets.

### Beneficial effect of polyclonal anti-Group II $PLA_2$ antibodies in rat endotoxin shock

*Giuseppe Cirino, Maria Rosaria Bucci and Carla Cicala*
Department of Experimental Pharmacology, via Domenico Montesano 49, 80131 Naples, Italy

Endotoxaemia leads to an increase in circulating levels of extracellular phospholipase $A_2$ in animal models as well as in patients with septic shock (Vadas *et al.*, 1992). We have tested rabbit polyclonal anti-$PLA_2$ antibodies raised against human recombinant secreted non pancreatic $PLA_2$ (Group II $PLA_2$) and porcine pancreatic $PLA_2$ (Group I $PLA_2$) in rat endotoxin shock. Fall in mean arterial blood pressure induced by endotoxin (25 mg/kg i.v.; serotype 0127:B8) was 48 ± 3 mmHg ($n = 13$) while for anti-snp-PLA (0.5 mg/kg) and anti-porc-$PLA_2$ (0.5 mg/kg) was 28 ± 3 mmHg ($n = 5$, $p < 0.01$) and 45 ± 10 mmHg ($n = 4$; ns). Anti-snp-$PLA_2$ at the lower dose tested (0.1 mg/kg) was ineffective while the inhibition achieved with the higher dose (1 mg/kg) was similar to that obtained with the dose of 0.5 mg/kg. From a preliminary study was observed that maximal reduction in leukocyte platelet and haematocrit was obtained 4 h after LPS administration. Polyclonal anti-hnps-PLA2 antibodies at the dose of 0.5 mg/kg prevented the change in platelet count but not in leukocytes and haematocrit (figure; the line indicates platelet control value).

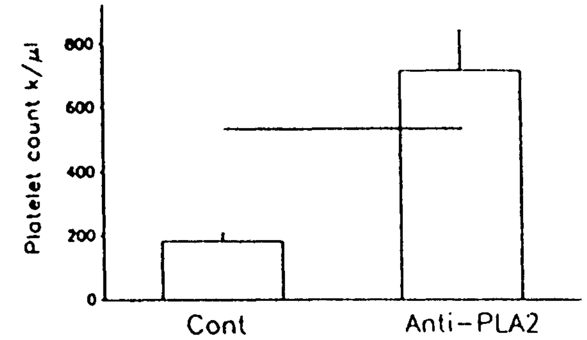

### Inhibitory effects of a mouse/human chimeric anti-TNFα antibody on *in vitro* immune function

*M. R. Dalesandro, C. S. Kinney, B. Frederick, B. Scallon and J. Ghrayeb*
Pharmaceutical Division, Centocor, Inc., Malvern, PA, USA

Tumour necrosis factor (TNF) α, a cytokine produced by activated macrophages and lymphocytes, is a known mediator of both normal and chronic inflammation. Properties of TNFα including stimulation of IL-1 and collagenase are consistent with participation in the pathogenesis of rheumatoid arthritis (RA). Elevated levels of TNFα are found in the synovial fluid and on the membranes of activated $CD4^+$ synoviocytes of RA patients. cA2, a mouse/human chimeric anti-TNFα antibody composed of the murine variable region joined to a human γ1 constant region, has shown clinical efficacy in the treatment of RA patients. Scatchard analyses reveal that cA2 γ1 and cA2 Fab bind human TNFα with equally high affinities and WEHI cytotoxicity assays show that they are equally efficient at inhibiting TNFα activity. *In vitro* assays showed that cA2 γ1 inhibited 60% of human peripheral blood cell proliferation in response to tetanus toxoid, mixed lymphocyte culture, or immobilized OKT3 antibody. The same molar concentration of cA2 Fab inhibited proliferation by a maximum of 25%. Similarly, cA2 γ1 inhibited T-B cell collaboration measured by Ig synthesis more effectively than did the cA2 Fab. Since affinities and neutralization of cytotoxicity are equivalent for the cA2 γ1 and Fab fragments, we are currently pursuing the possibility that the greater immunomodulation observed with cA2 γ1 reflects a combined ability to neutralize soluble TNFα and to promote Fc-mediated death of activated cells expressing membane-bound TNFα.

### Therapeutic intervention with mycobacterial 65 kDa heat-shock protein peptide 180-188 in adjuvant arthritis in Lewis rats

*Ulrich Feige and Jill Gasser*
Ciba-Geigy, Department of Inflammation, R-1056.1.84, CH-4002, Basel, Switzerland

Adjuvant arthritis (AA) in Lewis rats is induced by an injection of mycobacteria in oil. AA is a T-cell mediated disease. Mycobacterial 65 kDa heat shock protein (hsp65) has been shown to be one of the antigens involved in the disease process. In fact, hsp65 or peptide 180–188 of hsp65 can be used to protect rats against an arthritogenic challenge with mycobacteria. However, therapy rather than prevention of disease appears to be the appropriate goal for intervention with disease in man. Therefore, we investigated whether peptide 180–188 also exhibits therapeutic effects in AA. We found that treatment of rats with 0.1–1.0 mg of peptide 180–188 in incomplete Freund's adjuvant i.p. at days 9 and 10 after the arthritogenic challenge with mycobacteria in oil completely inhibited the onset of AA (no paw swelling, weight loss or radiographical changes were present). Even treatment at days 12 and 13, when clinical symptoms of disease (paw swelling and weight loss) were already apparent, was effective. The extent of bone destruction was reduced; individual radiographical scores at day 35 were 0, 0, 15, 72, 78 in peptide 180–188 treated rats and 44, 47, 57, 75, 101, 119 in AA control rats. The data indicate that immunological intervention with peptide 180–188 brings the disease process to a halt. This suggests that even in established disease the immune response is the major driving force of the disease process.

### Protection of human endothelial cells from the cytotoxic effects of activated granulocytes by Lazaroids and related lipophilic antioxidants

*William E. Fleming and Frank F. Sun*
Hypersensitivity Diseases Research, Upjohn Labs, Kalamazoo, MI 49001, USA

The damaging of vascular endothelium by activated granulocytes is a typical feature of the inflammatory response. *In vitro* human granulocytes (neutrophils or eosinophils) activated by phorbol myristyl acetate (PMA) can injure and kill human umbilical vein endothelial cells (HUVECs). We incubated PMA activated human granulocytes with $^{51}$-chromium labelled HUVECs for 4 h and determined the extent of cell injury by measuring the release of radioactivity into the medium. The results confirmed previous findings that granulocyte-induced cytotoxicity is iron and passage dependent with respect to the endothelial cells. Since it was postulated that the granulocyte derived reactive oxygen species were the predominant cytotoxic agents, we examined the effects of lipophilic antioxidants such as the lazaroid U-74006F, vitamin E, the vitamin E analogue U-78517F, the phenolic antioxidant nordihydroguaiaretic acid, the iron chelator desferroximine, the antiinflammatory steroids and the water soluble antioxidant pyrrolidine dithiocarbamate (PDTC) as potential inhibitors of granulocyte induced endothelial cell cytotoxicity. As expected, the cytotoxicity was blocked by appropriate concentrations of lipophilic antioxidants. The results suggest that lipophilic antioxidants can protect against granulocyte-induced endothelial cell injury during inflammation.

### Zymosan induced arthritis in rats: pharmacological sensitivity

*P. Gegout, P. Gillet, D. Chevrier, C. Guingamp, B. Terlain and P. Netter*
Department of Pharmacology, URA CNRS 1288, BP184, 54505 Nancy, France

Zymosan, a glycan derived from yeast cell wall is a phlogistic agent activating the alternative pathway of the complement system and inducing lysosomal enzyme release from PMNs and macrophages. Since zymosan-induced arthritis was mainly studied in mice, we have investigated its course in rats and assessed its inflammatory profile. Zymosan (2 mg) was injected (on day 0) in the right (histological and pharmacological assessment) or both knee joints (actimetry) Studies of patellar cartilage metabolism were assessed *ex vivo* through proteoglycan content and $^{35}$S incorporation.

After injection of zymosan, swelling of right injected joints reached a maximum after 24 h and then decreased. Functional impairment was maximal the night following i.a. injection, and then decreased progressively. Histologically, the arthritis was characterized at day 14 by a chronic proliferative synovitis eroding cartilage. After an initial fall of chondrocyte proteoglycan synthesis peaking on day 2, occurred a transitory resumption on day 4. On day 20, anabolism of proteoglycans seemed normal, but proteoglycan content in arthritic patellae was significantly reduced (−18.6%).

Indomethacin (IND), when given orally from day-1, was effective on knee swelling only on day 1 and at high dose (3 mg/kg/day) while dexamethasone (DEX, 0.1 and 0.5 mg/kg/day) significantly reduced knee swelling through the first week. Histological score and locomotor activity were not improved by IND. In contrast, DEX

significantly reduced histological lesions. The initial fall of cartilage anabolism was worsened by IND (3 mg/kg). On the other hand, IND was without influence on reduced proteoglycan content.

In summary, with regard to its drug sensitivity, it is likely that zymosan induced synovitis in rats results only partly from cyclooxygenase activation and mostly from other phospholipids pathways, i.e. 5-LO, PAF... Thus, this model of subacute synovitis behaves in a similar manner to rheumatoid arthritis and seems potentially useful to evaluate anti-inflammatory drugs and potentially antidegenerative (chondroprotective) drugs.

### Role of nitric oxide in the pathophysiology of pancreas inflammation

*E. Gelpí, G. Hotter, D. Closa and J. Roselló-Catafau*

Molecular Pathology Unit, Dpto. Bioanalítica Médica, Centro de Investigación y Desarrollo, CSIC, Barcelona, Spain

The relationship between nitric oxide production and prostanoid generation in two pancreas inflammatory process: ischaemia-reperfusion in pancreas transplantation and pancreatitis has been studied. For this purpose male Sprague–Dawley rats were subjected to pancreas transplantation, after 12 h preservation or acute pancreatitis induced by intraductal administration of 3.5% sodium taurocholate. In both cases, the effect of nitric oxide synthase inhibition with administration of $N^G$-nitro-L-arginine methyl esther (L-NAME) (10 mg/kg) was tested. The results show increases in 6-keto $PGF_{1\alpha}$, $TXB_2$ and $PGE_2$ in pancreatic tissue after transplantation or pancreatitis. In the case of transplantation, nitric oxide synthase inhibition reversed all the observed increases, suggesting that eicosanoid generation in this process would be mediated in part by a nitric oxide dependent mechanism. In contrast, in pancreatitis, nitric oxide synthase inhibition only reversed the increases in 6-keto $PGF_{1\alpha}$ and $TXB_2$ levels, suggesting that endothelial and platelet eicosanoid generation are mediated through an nitric oxide-dependent mechanism, while acinar metabolites ($PGE_2$) are generated in response to cell damage.

### Effects of endotoxin (LPS) on carbon-tetrachloride intoxicated rats

*J. Gergely,[1] S. Sipka,[2] M. Udvardy,[3] A. Kulcsár,[3] Sz. Gomba,[4] I. Csipő[2] and Gy. Szegedi[2]*

[1]Department of Pharmacology, [2]3rd Department of Internal Medicine, [3]2nd Department of Internal Medicine, [4]Department of Pathology, University Medical School of Debrecen, Hungary

We have measured the effects of *E. coli* lipopolysaccharide (LPS: 40 μg/kg) in two groups of male Wistar rats intoxicated with a single dose of carbontetrachloride (CCL: 125 ml/kg). In group 1 LPS was applied 1 h, in group II 24 h after $CCl_4$ treatments. Lethality (25%) was found only in group II. Compared with $CCl_4$, additional LPS resulted in still further increases both in respiratory frequency and heart beat/min. In $CCl_4$ treated animals we observed an elevation in serum prostacyclin levels which could be related to the high amount of circulating $PLA_2$ measured. In the 24th hour of $CCl_4$ intoxication in the phase of progressed damage of liver, rats were more sensitive to the effect of LPS than in an earlier phase in the first hour of injury.

We suppose that in $CCl_4$ intoxicated persons the increased sensitivity to endotoxin can be one of the factors of clinical complications.

### Antipyretic properties of paracetamol in normal rats and during various models of inflammation

*P. Gillet, I. Cherkaoui, P. Gegout, F. Lapicque, E. Boccard,* B. Terlain and P. Netter*
Department de Pharmacologie, URA CNRS 1288, BP184, F54505, Nancy; *Laboratoires UPSA, F92500 Rueil Malmaison, France

Paracetamol is a widely used antipyretic-analgesic drug, which inhibits cyclooxygenase (COX). Recent data have suggested the difference of sensitivity of COX, peripheral or central, constitutive (COX1) or inducible (COX2) to paracetamol. We have investigated the impact of paracetamol on body temperature (biotelemetry devices) in normal rats and during prostaglandin-dependent models of synovitis and fever. Paracetamol was injected i.p. (25; 50; 100; 200 mg/kg) through its prodrug, propacetamol (PRODAFALGAN).

In normal rat, body temperature and activity vary on a circadian basis. In this nocturnal animal, body temperature is highest at night and lowest during the day, mainly mediated by prostaglandins (PG). During day-time the highest dose of paracetamol (200 mg/kg) resulted in a slight decrease in body temperature with a maximal effect ($E_{max}$:–0.61°C) observed a $T_{max}$ of 1.75 h. During night-time, the 200 mg/kg dose only induced a fall in body temperature ($E_{max}$:–1.06°C; $T_{max}$: 1.5 h).

Injections of carrageenan in rat knees induced an acute fever (39°C) and nociceptive-induced hypomobility (–60%). Paracetamol prevented transiently the febrile response and induced a dose-dependent fall in body temperature. With the 200 mg/kg dose $E_{max}$ (–1.27°C) was observed after a $T_{max}$ of 2 h with an amplitude *vs* control batch of about –2.5°C. In contrast, paracetamol did not restore mobility in arthritic rats. Paracetamol administered in an established yeast-induced fever demonstrated dose-dependent antipyretic properties, maximal with the 200 mg/kg dose ($E_{max}$: –3.11°C; $T_{max}$: 2.5 h).

These results demonstrate hypothermic properties of high doses of paracetamol in normal rats, and antipyretic properties at therapeutic regimen in inflamed rats. The magnitude of maximal effect and duration of antipyretic properties appears related to the degree of activation of inducible COX. In addition central properties of paracetamol are more marked on central PG (fever) than on peripheral PG (synovitis with hypomobility).

### Ultraviolet B-induced inflammatory cytokine production, *in vivo*: initial pharmacological characterization

*D. E. Griswold and M. N. Tzimas*
Department of Inflammation and Respiratory Pharmacology, SmithKline Beecham Pharmaceuticals, King of Prussia, PA 19406-0939, USA

Tumour necrosis factor alpha (TNFα) and inflammatory cell infiltration following ultraviolet B (UVB) irradiation was studied. Balb/c mice were exposed to UVB irradiation (10–30 min) using a bank of six Westinghouse FS40 sunlamps (emitting a spectrum from 270–320 nm; 65% in UVB range). Energy output was 1067 $\mu W/cm^2$. TNFα was measured in tissue homogenates using an EIA. Myeloperoxidase (MPO) enzyme activity was used as a marker of neutrophil infiltration. A timecourse and dose-response of UVB revealed a delayed onset of response for both TNFα and MPO. Administration of naproxen (20 mg/kg, p.o.) appeared to exaggerate the response to UVB (122% increase of TNFα, $p < 0.01$; 15% increase of MPO, n.s.). In contrast, the phosphodiesterase inhibitor, rolipram (10 mg/kg, p.o.) significantly inhibited the release of TNFα (78%, $p < 0.001$) and the MPO response (61%, $p < 0.001$). This initial characterization of UVB-induced inflammation suggests a relationship between TNFα release and inflammatory cell infiltration and strong regulation of these events by cyclic AMP.

### The modulation of adhesion molecule expression on endothelial cells by acteoside, a component of *Stachs Sieboldii* MI'Q

*K. Hayashi, T. Nagamatsu and Y. Suzuki*
Meijo University, Nagoya 468, Japan

Recently, it has been indicated that adhesion of leukocytes to endothelial cells play the important role in both immune and inflammation response. The development of agent which is able to blunt the adhesion will be very beneficial to treat a lot of inflammatory diseases. Acteoside (ACT) inhibited the adhesion of neutrophils to cultured human umbilical vein endothelial cells (HUVEC) stimulated with tumour necrosis factor (TNF)α and up-regulation of ICAM-1 expression, but not endothelial leukocyte adhesion molecule-1 on HUVEC, with TNFα, interleukin-1β or phorbol myristate acetate. The inhibitory effect was not due to the toxicity of acteoside to HUVEC. On the other hand, ACT did not increase the amount of soluble ICAM-1 in the culture medium of HUVEC treated by TNFα, nor affected the interaction of ICAM-1 and BBIG-11 (anti-human ICAM-1 monoclonal antibody). The present data suggest that ACT is promising as an anti-inflammatory agent in a new category. Now, we are investigating the effect of ACT on ICAM-1 expression by thrombin which do not mediate the *de novo* synthesis of ICAM-1.

### Effects of BIRM 270 on neutrophil functions

*A. F. Hoffman, A. G. Graham, J. Watrous, C. A. Homon, E. David, R. P. DeLeon, R. M. Dinallo, G. Hansen, E. Mainolfi, R. Rothelein, P. R. Farina and T. P. Parks*
Departments of Biochemistry, Analytical Chemistry and Immunology, Boehringer Ingelheim Pharmaceuticals, Ridgefield City, CT, USA

Neutrophils are phagocytes which play a major role in host defence by emigrating from the blood compartment into sites of injury or infection where they release proinflammatory mediators and bactericidal agents. Recently, we identified a compound, BIRM 270, which potently inhibited the biosynthesis of $LTB_4$ by calcium ionophore A23187-stimulated neutrophils. The compound was found to act by blocking the release of arachidonic acid. The present investigation was conducted to further define the effects of BIRM 270 on lipid mediator biosynthesis and various other neutrophil functions, some previously linked to arachidonate mobilization. Ionophore-stimulated arachidonate release and platelet activating factor biosynthesis (PAF), quantified by GC/MS, were inhibited similarly by BIRM 270, with > 90% inhibition achieved at 30 nM and half-maximal inhibition at about 14 nM. BIRM 270 did not affect any other neutrophil responses investigated, including: chemotaxis; Mac-1 expression (from mobilization of secretory vesicles); adherence to activated endothelium; lysozyme release (from degranulation of specific and azurophilic granules); lactoferrin release (from specific granules); or the generation of superoxide radical, as measured by the reduction of cytochrome C in the presence or absence of superoxide dismutase. We conclude that the actions of BIRM 270 are restricted to the biosynthesis of lipid mediators, and that arachidonate may not play an important second messenger role in other neutrophil functions, e.g. NADPH oxidase activation. In addition, our results provide pharmacological evidence that arachidonate mobilization and PAF biosynthesis may be coordinately linked.

### Methyl arachidonyl fluorophosphonate, a potent irreversible $cPLA_2$ inhibitor, blocks the mobilization of arachidonic acid in human platelets and neutrophils

*Z. Huang, S. Liu. I. Street, F. Laliberte, K. Abdullah, S. Desmarais, Z. Wang, B. Kennedy, P. Payette, D. Riendeau, P. Weech and M. Gresser*
Merck Frosst Centre for Therapeutic Research. P.O. Box 1005, Pointe-Claire, Dorval, Quebec, Canada, H9R 4P8

Methyl arachidonyl fluorophosphonate (MAFP) was designed as a $cPLA_2$ inhibitor based on its preference for arachidonyl moiety and the possible involvement of a serine residue in its active site. MAFP wax found to be a potent time-dependent irreversible inhibitor of $cPLA_2$. It inhibited $cPLA_2$ stoichiometrically *in vitro* with an estimated $k_{on}$ of 0.9 $s^{-1}$ mol fraction$^{-1}$ on the 4 mol% DTEPM/ Triton X-100 mixed micellar interface in the presence of calcium. Preincubation of $cPLA_2$ with the reversible inhibitor $AACOCF_3$ (arachidonyl trifluoromethyl ketone) protected $cPLA_2$ from MAFP, indicating $AACOCF_3$ competes with MAFP. Whereas the human secreted 14-kDA phospholipase $A_2$ ($sPLA_2$) was not inhibited by MAFP at concentration as high as 10 mol%. Thus, MAFP is a potent selective active-site directed inhibitor of $cPLA_2$. MAFP was not an inhibitor for human 5-lipoxygenase when tested at 10 μM (~ 30 mol%) *in vitro*; however, it inhibited the A23187-induced production of $LTB_4$ in human neutrophils with a $IC_{50}$ of ~ 0.1 μM. Furthermore, MAFP blocked the A23187-induced AA release with a $IC_{50}$ of 0.6 μM in the presence of ETYA in human platelet. MAFP also inhibited A23817-induced 12-HETE ($IC_{50}$ 0.6 μM) and $TxB_2$ ($IC_{50}$ 0.6 μM) production by platelets. In contrast, it did not inhibit 12-HETE and $TxB_2$ production in platelets when exogenous AA was used as a stimulus instead of A23187 ($IC_{50}$ > 10 μM). These results indicate that MAFP is a potent inhibitor capable of blocking the production of AA in neutrophils and platelets. $cPLA_2$ might be involved in A23817-induced mobilization of AA in neutrophils and platelets. The identical $IC_{50}$s for inhibition of AA, $TxB_2$ and 12-HETE production in platelets suggest that $TxB_2$ and 12-HETE were derived from the same pool of AA.

### Platelet-derived growth factor (PDGF) and angiotensin II (A II) stimulate the mitogen-activated protein kinase cascade in renal mesangial cells

*A. Huwiler,*[1] *S. Stabel,*[2] *D. Fabbro*[3] *and J. Pfeilschifter*[1]

[1]Department of Pharmacology, Biozentrum, Switzerland; [2]Max-Delbrück-Labor, Köln, Germany; [3]Pharmaceuticals Division, Ciba-Geigy Ltd, Basel, Switzerland

Treatment of mesangial cells with PDGF and A II rapidly and dose-dependently stimulated mitogen-activated protein (MAP) kinase activity. Whereas stimulation with PDGF-BB caused a potent and sustained (for more than 30 min) phosphorylation and activation of $p42^{mapk}$ and $p44^{mapk}$ as well as of the upstream activators MAP kinase (MEK) and c-Raf, the effect of A II was less potent and peaked at 5–10 min and thereafter declined rapidly.

Down-regulation of protein kinase C (PKC)-α and -δ isoenzymes by 4 h or 8 h treatment with phorbol 12-myristate 13-acetate (PMA) markedly inhibited PDGF-induced MAP kinase activation. Exposure to PMA for 24 h, a regimen that also depletes PKC-ε, did not further reduce the level of activation by PDGF-BB, suggesting that both PKC-dependent and PKC-independent pathways are involved in PDGF-stimulated MAP kinase activation. A II-stimulated MAP kinase activity was decreased by 60% after 8 h of PMA treatment, a time period that caused complete down-regulation of PKC-α and -δ. In contrast to PDGF a 24 h treatment with PMA completely abolished A II-stimulated MAP kinase activity indicating that PKC-ε substantially contributes to A II-stimulation of the MAP kinase pathway. Activators of protein kinase A, such as forskolin or $N^6$, 2′-O-dibutyryl-cAMP, attenuated PDGF- and A II-stimulated MAP kinase activation.

In summary, these results suggest that PDGF-BB and angiotensin II differ in their potency and duration of activating of the MAP kinase cascade which may explain why PDGF-BB is a potent mitogen for mesangial cells whereas A II only triggers mesangial cell hypertrophy.

### Regulation and expression of prostaglandin G/H synthase in U937 cells

*J. J. Johnston and J. M. Trzaskos*
The DuPont Merck Pharmaceutical Company, Inflammatory Diseases Research, P.O. Box 80400, Wilmingtòn, DE 19880-0400, USA

Prostaglandin G/H synthase catalyses the rate limiting step in the production of prostaglandins from arachidonic acid. Inhibition of this enzyme by non-steroidal anti-inflammatory drugs (NSAIDs) has proven useful in the treatment of inflammatory conditions, however, mechanism-based GI and renal side effects are observed with most NSAIDs. Recently, an inducible form of PGHS has been discovered which is thought to be the major contributor to prostaglandin production during inflammation. In order to more completely define the contribution of this isoform to inflammation, an understanding of the expression patterns of both PGHS genes is necessary. We have shown that numerous agents including serum, TPA, LPS, and TNF-α can induce the expression of PGHS2 mRNA in fibroblasts while having minimal effects on PGHS1 expression. Using luciferase reporter gene constructs containing between 600 bp and 2 kb of the murine PGHS2 promoter region, luciferase activity can be induced by serum, while TPA, IL-1 and TNF-α do not show an effect. When these reporter gene constructs are transfected into the monocytic U937 cell line they are responsive to TPA, showing a ten-fold increase in luciferase activity upon TPA stimulation. The inability of TPA to act through the promoter region in fibroblasts, coupled with the lack of clear AP-1 elements in this region of PGHS2 suggests that a monocyte specific factor may be responsible for the TPA response seen in U937 cells.

### Tetrahydrobiopterin is a limiting factor of nitric oxide generation in interleukin 1β-stimulated rat glomerular mesangial cells

*H. Mühl and J. Pfeilschifter*
Department of Pharmacology, Biozentrum, University of Basel, Basel, Switzerland

Treatment of mesangial cells with recombinant human interleukin 1β (IL-1β) triggers the expression of a macrophage-type of nitric oxide (NO) synthase and the subsequent increase of cellular concentration of cGMP and nitrite production. Tetrahydrobiopterin (BH4) is an essential cofactor for NO synthase and in the present study we investigated its impact on inducible NO synthesis in mesangial cells. Inhibition of GTP-cyclohydrolase I, the rate-limiting enzyme for BH4 synthesis, with 2,4-diamino-6-hydroxy-pyrimidine (DAHP) potently suppresses IL-1β-induced nitrite production and elevation of cellular cGMP levels. This inhibitory effect of DAHP is reversed by sepiapterin, which provided BH4 via the pterin salvage pathway. Most importantly, sepiapterin dose-dependently augments IL-1β-stimulated NO synthesis, indicating that the availability of BH4 limits the production of NO in cytokine-induced mesangial cells. *N*-acetylserotonin, an inhibitor of the BG4 synthetic enzyme sepiapterin reductase, completely abolishes IL-1β-stimulated nitrite production, whereas methotrexate, which inhibits the pterin salvage pathway, displays only a moderate inhibitory effect, thus suggesting that mesangial cells predominantly synthesize BH4 by *de novo* synthesis from GTP. In conclusion, these data demonstrate that BH4 synthesis is an absolute requirement for, and limits IL-1 β induction of NO synthesis in mesangial cells. Inhibition of BH4 synthesis may provide new therapeutic approaches to the treatment of pathological conditions involving increased NO formation.

### Anti-inflammatory effect of BMS-181162, a $PLA_2$ inhibitor on two animal models of immune modulated inflammation

*X. Nair, L. Davern, C. Gabrel, P. Stanley and K. Tramposch*
Dermatology Research, Buffalo, NY, USA

BMS-181162, a known inhibitor of $PLA_2$, is active against models of immune modulated inflammation, viz. The reverse passive arthus (RPA) model in rats and the delayed type hypersensitivity model in mice (DTH). The inflammatory response in the RPA model is precipitated by immune complex deposition, and complement-fixation, with PMN infiltration, and vascular permeability increase. Vascular permeability was measured by the accumulation of $^{125}$I-BSA in tissue and PMN infiltration was measured by myeloperoxidase (MPO) activity in the reaction site. BMS-181162 in a Tween 80/normal saline (0.5/99.5) vehicle was dosed i.p. 2 h before the administration of anti-BSA and BSA. Drug effect was measured 4 h post antigen/antibody administration. The DTH reaction in mice was induced by topical application of oxazolone. BMS-181162 was applied topically to the ear and PMN infiltration was measured by MPO activity. In the RPA model BMS-181162 caused a dose-dependent inhibition of vascular permeability and PMN infiltration. BMS-181162 at 50 mg/kg also significantly inhibited $PGE_2$ and $LTB_4$ increases. In the DTH assay BMS-181162 (2%) reduced PMN infiltration in the inflamed ear by 63%. It is significant that this $PLA_2$ inhibitor has demonstrated activity against two models of immune modulated inflammation following systemic and local administration. These results suggest the potential for developing agents that are active against both immune complex and cell mediated inflammation.

### Demonstration and characterization of anti-inflammatory activity in a carbohydrate (glycogen) extract of the New Zealand green-lipped mussel

*D. J. Ormrod, J. R. Dodd, R. Geddes[1] and T. E. Miller*
Departments of Medicine and [1]Biochemistry, University of Auckland, Auckland, New Zealand

Many effective modern drugs were originally derived from plants or animals. In the past two decades there has been renewed interest in the search for pharmaceuticals in nature. The marine environment, with its huge and diverse reservoir of species, has received particular attention. Many potentially effective compounds have been isolated, but few have been used clinically. However, in cases where the parent organism can be economically harvested or farmed, nutraceuticals with apparent clinical benefits have reached the marketplace. Freeze-dried NZ green-lipped mussel (*Perna canaliculus*), marketed internationally as Seatone©, is one example (McFarlane Laboratories, Auckland, New Zealand). Laboratory and clinical investigations have shown that this preparation inhibits experimentally induced inflammation and may provide symptomatic relief in individuals with arthritis. We have studied the mollusc in an effort to identify the active moiety. Our most recent results demonstrate that the activity resides in a glycogen fraction of the mussel. The extract effectively reduces carrageenin-induced inflammation in rats, following either oral or parenteral administration. The compound also suppresses neutrophil emigration, possibly by blocking carbohydrate receptors on neutrophils or endothelial cells.

### Transcriptional activation of lipocortin-1

*L. Parente, F. Russo-Marie[1] and E. Solito*

I.R.I.S. Siena, Italy and [1]I.C.G.M. Inserm U332, Paris, France

Lipocortin-1 (annexin-1) is a member of a family of proteins with calcium and phospholipid binding properties which is a putative mediator of the anti-inflammatory action of glucocorticoids. Since controversial data have been reported on the capability of steroids of inducing the synthesis of lipocortin-1 we have studied the promoter region of 880 bp of the protein gene which contains the first not translated exon and the first intron. Different cell lines (HeLa, U-937, A-549) wee transfected with a plasmid containing the luciferase reporter gene under the control of the lipocortin-1 promoter. Transient expression of the reporter gene was enhanced by phorbol 12-myristate 13-acetate (PMA, 10 nM), dexamethasone (DEX, 1 μM), and IL-1 (500 pg/ml). Interestingly the stimulatory effect of PMA was counteracted by a short incubation (2 h) with DEX while it was potentiated by a long treatment (16 h) with the glucocorticoid. These results indicate that glucocorticoids are indeed able to enhance gene expression of lipocortin-1. The inhibitory effect of 2 h DEX on PMA-stimulated gene expression may be explained by interference with AP-1 activity, which is induced by PMA, by direct intereaction or by DNA binding. The synergistic effect of 16 h DEX may be due to phosphorylation by PMA of transcription factors interacting with the glucocorticoid receptor.

### Detection of soluble ICAM-1, cytokines (IL-1β, IL-6, TNFα) and eicosanoids ($LTB_4$, $PGE_2$, $TXB_2$, $6kPGF_{1\alpha}$) in ascites of patients with portal hypertension, peritoneal cancer and spontaneous bacterial peritonitis

*W. M. Pruimboom, D. J. Bac, I. L. Bonta, J. H. P. Wilson and F. J. Zijlstra*
Pharmacology and Internal Medicine, Erasmus University, Rotterdam, The Netherlands

Ascites is a complication of portal hypertension and of peritoneal cancer. Spontaneous bacterial peritonitis (SBP) is a serious complication in these patients. We measured soluble ICAM-1 (sICAM-1), several cytokines (interleukin-1β (IL-1β), IL-6 and tumour necrosis factor-α (TNF-α)) and several eicosanoids ($LTB_4$, $PGE_2$, $TXB_2$ and $6kPGF_{1\alpha}$) in ascites and serum from patients (54) with cirrhosis (alcoholic, viral, PBC and other causes), peritoneal cancer (26) and SBP (10) to determine whether the pattern of mediators found could be of diagnostic and/or prognostic aid.

Ascitic sICAM-1, the cytokine IL-6 and the eicosanoids $LTB_4$ and $PGE_2$ were significantly elevated in peritoneal cancer compared with portal hypertension. Ascitic IL-6 was significantly elevated in SBP compared with portal hypertension and peritoneal cancer. There was a positive correlation ($p < 0.001$) between serum and ascites sICAM-1 levels, but not for the other metabolites. During SBP there was a significant increase in ascitic sICAM-1 and IL-6 (not in serum), which rapidly declined after antibiotic treatment.

Conclusion: There are significant differences in concentrations of inflammatory products in ascites due to different disorders, but none of these mediators seems to be useful as a diagnostic parameter as there is an overlap between all the levels of these mediators in ascites. SICAM-1 and IL-6 could be of prognostic aid for patients with SBP, as the episodes of infection correlate with the levels of these mediators.

### Syndetoxin[R] binds phospholipase $A_2$, tumour necrosis factor and endotoxin, and significantly decreases the lethality of endotoxin shock in rats

*S. Sipka,[1] G. Bot,[2] P. Gergely, L. Bali,[4] P. Sápy,[5] J. Csongor,[3] L. Bertók[6] and G. Szegedi[1]*

[1]3rd Department of Internal Medicine, [2]Department of Medical Chemistry, [3]Central Isotope Laboratory, [4]2nd Department of Surgery, University of Debrecen, [5]TRIGON Laboratory, [6]National Research Institute for Radiology, Budapest, Hungary

Syndetoxin[R] (SDTX) is a semisynthetic, non toxic biopolymer which dose-dependently binds $^{125}I$-labelled human secretable phospholipase $A_2$ (14 KD), $^{125}I$-labelled human recombinant tumour necrosis factor and $^{99m}Tc$ labelled *E. coli* lipopolysaccharide (endotoxin). 10 mg/kg i.v. pretreatment of rats with SDTX result in a 60% survival of animals sensitized by Pb acetate for the 100% lethal dose of endotoxin. SDTX has still an antiaggregatory effect on human platelets by binding of aggregating agents, e.g. platelet activating factor or ADP.

The mechanism of Syndetoxin[R] action is the simultaneous binding, absorption of several types of toxic agents or toxins. This character of SDTX can be very advantageous especially.in septic or other types of shock where lots of toxic substances are produced and sustaining the pathologic state.

### Pharmacologic efficacy of LY293111, a potent orally active leukotriene $B_4$ ($LTB_4$) receptor antagonist, in humans

*S. M. Spaethe,[1] P. Marder,[1] L. L. Froelich,[1] B. H. Petersen,[1] T. W. Croghan,[1] R. A. Lucas,[2] T. Tanner[3] and J. S. Sawyer[1]*

[1]Lilly Research Laboratories, Indianapolis, IN, USA; [2]Lilly Research Centre, Windlesham, UK; [3]Simbec Research Ltd, Merthyr Tydfil, South Wales, UK

A common element of inflammation is the accumulation and activation of leukocytes at inflammatory foci. These events are directed by biological mediators, including $LTB_4$. Among the various physiological changes that neutrophils (PMNs) undergo during activation is an increase in the number of the adhesion receptors, CD11b/CD18, on the cell surface as measured by flow cytometry. Pre-incubation of human PMNs, either isolated or residing in whole blood with LY293111 prevents the subsequent upregulation of CD11b in response to $LTB_4$. We applied this technique to evaluate the pharmacodynamic profile of LY293111 in man. Volunteers received either LY293111 or placebo orally, and sequential blood samples were taken and challenged *ex vivo* with $LTB_4$ (10 nM). The degree of CD11b receptor upregulation was measured over time for each subject. There was marked inhibition (>73%) of $LTB_4$-induced CD11b expression on whole blood PMNs from volunteers receiving either 60 or 120 mg t.i.d., or 200 mg b.i.d. 8–12 h after dosing. This inhibition was reversible upon termination of dosing. Additional studies demonstrated the selectivity of LY293111 utilizing an alternative agonist, fMLP, in these same subjects following the high dose of LY293111. We conclude that LY293111 has potent, selective pharmacologic activity in man. These observations could provide the basis for novel therapeutic treatments of human inflammatory diseases.

### Activation of human eosinophils by cytokines: differential expression of adhesion/activation markers and potentiation of survival by chemokines

*G. E. Winterrowd, F. F. Sun, C. A. Hatfield, W. E. Fleming, N. J. Crittenden, I. M. Richards and J. E. Chin*
Upjohn Laboratories, Kalamazoo, MI 49001, USA

We have examined by flow cytometric analysis the differential induction of a panel of adhesion/activation markers on normal human peripheral blood eosinophils by IL-3, IL-5 and GM-CSF after 18 h and 48 h of *in vitro* culture. The expression of CD11a, CD11b, CD11c and CD18 were increased 18 h and 48 h after activation by all three cytokines at 10 ng/ml. Of the cytokines tested, IL-3 had the most pronounced effect when compared with IL-5 and GM-CSF. CD49b, CD49e and CD49f were found on very few of the inactivated eosinophils, but CD49d and CD29 were present on >90% of these cells. Expression of the β1 integrins remained unchanged even after 48 h of incubation. All of the eosinophils expressed CD44, CD58 (LFA-3), CD45RO, CD67, CD9, CD63 and CD31 but the mean channel fluorescence (MCF) of these antigens (except for CD31 and CD63) was elevated by IL-3, IL-5 and GM-CSF. The percentage of eosinophils which expressed CD25, CD69 and CD54, and the MCF were both increased by these cytokines. Conversely, the expression of L-selectin was dramatically reduced by cytokine treatment (IL-3>>IL-5=GM-CSF). RANTES, MCP-1, or MIP-1α/β (all at 20 ng/ml) did not alter the expression of any of the eosinophil cell surface antigens. Furthermore, these chemokines did not potentiate the effect of IL-3 or IL-5. RANTES, MCP-1, or MIP-1α/β alone did not prolong the survival of eosinophils even when the cells were placed on laminin, Collagen Type I or IV, and fibronectin-coated plates. However, the prolongation of eosinophil survival by IL-3 was enhanced by the addition of chemokines. Potential cytokine and chemokine interactions may contribute to the pathophysiology of eosinophilic inflammation.

### Involvement of CD36 in acute lung inflammation and injury in IgG immune complex-mediated rat lung injury

*Z.-J. Yang, C. Black and B. Beaubien*
Allelix Biopharmaceuticals Inc., Mississauga, Ontario, Canada L4V 1V7

CD36 is a cell surface receptor for thrombospondin (TSP), and has been determined to mediate significant effects of TSP on cell adhesion and platelet aggregation. Since elevated TSP levels have also been demonstrated in inflammatory sites (*J Immunol* 1989; **143**: 1969–73), we investigated the role of CD36 in acute lung inflammation using a rat model of IgG immune complex-induced alveolitis (*J Clin Invest* 1974; **54**: 349–357). To generate the immune response, rats were given bovine serum albumin (BSA) intravenously and anti-BSA IgG intratracheally. Four hours later, the following parameters were measured: total cell numbers, cell differentials, haemoglobin concentrations in bronchoalveolar lavage (BAL), plasma extravasation in BAL, and plasma exudation in perfused lungs. Monoclonal antibodies (Mabs) directed against human CD36 (OKM5 and MCA722) induced a dose-dependent inhibition of airway leukocyte infiltration, haemorrhage and plasma exudation. The two Mabs were approximately equipotent. Intratracheal administration of the same dose of Mab was more effective than an intravenous dose. Isotype control immunoglobulins had no effect. These findings demonstrate for the first time that CD36 is involved in lung damage in this model.
